# The Rise of Passive RFID RTLS Solutions in Industry 5.0

**DOI:** 10.3390/s24051711

**Published:** 2024-03-06

**Authors:** Ygal Bendavid, Samad Rostampour, Yacine Berrabah, Nasour Bagheri, Masoumeh Safkhani

**Affiliations:** 1 Department of Analytics Operations and Technology, School of Management, Université du Québec à Montréal (UQAM), Montreal, QC H2X 3X2, Canada; 2 Department of Computer Science, Vanier College, Montreal, QC H4L 3X9, Canada; rostamps@vaniercollege.qc.ca; 3 CPS^2^ Lab, Electrical Engineering Department, Shahid Rajaee Teacher Training University, Tehran 16788-15811, Iran; nbagheri@sru.ac.ir; 4 Computer Engineering Department, Shahid Rajaee Teacher Training University (SRTTU), Tehran 16788-15811, Iran; safkhani@sru.ac.ir

**Keywords:** IoT, passive UHF RFID, RTLS, real-time location systems, IPS, indoor positioning systems, industry 5.0, digital transformation

## Abstract

In today’s competitive landscape, manufacturing companies must embrace digital transformation. This study asserts that integrating Internet of Things (IoT) technologies for the deployment of real-time location systems (RTLS) is crucial for better monitoring of critical assets. Despite the challenge of selecting the right technology for specific needs from a wide range of indoor RTLS options, this study provides a solution to assist manufacturing companies in exploring and implementing IoT technologies for their RTLS needs. The current academic literature has not adequately addressed this industrial reality. This paper assesses the potential of Passive UHF RFID-RTLS in Industry 5.0, addressing the confusion caused by the emergence of new ’passive’ RFID solutions that compete with established ’active’ solutions. Our research aims to clarify the real-world performance of passive RTLS solutions and propose an updated classification of RTLS systems in the academic literature. We have thoroughly reviewed both the academic and industry literature to remain up to date with the latest market advancements. Passive UHF RFID has been proven to be a valuable addition to the RTLS domain, capable of addressing certain challenges. This has been demonstrated through the successful implementation in two industrial sites, each with different types of tagged objects.

## 1. Introduction

Beginning in the 2000s, the North American manufacturing sector gradually lost significance. This decline in manufacturing activity was driven by various factors, including growth in service industries, alongside strong competition from Asian countries, which leverage cheaper labor and extensive production expertise. In addition to these combined effects, there was a lack of investment in technology by manufacturing companies. To compound matters, the COVID-19 pandemic dealt a severe blow to the sector in 2020.

To address this sectoral weakness, stimulate manufacturing, enhance productivity, and support competitiveness, governments worldwide proposed digital economy action plans. These plans underscored the crucial need for modernization and adoption of Industry 4.0/5.0 concepts [1], often referred to as Industry X.0, as coined by Schaeffer in 2017 [2].

While strategically, digital transformation forces companies to reassess their business models and management approaches, practically, many companies start this journey by trying to solve operational problems [3]. Indeed, an initial focused project often serves as a gateway for companies motivated by practical business concerns, rather than a shift in mindset towards services, value, and outcomes. As Ferreira et al. [4] point out, SMEs are increasingly interested in moving towards Industry 4.0, whether driven by internal motivation or by pressure (from customers and/or large companies), especially if the project aims at cost reduction and short-term benefits (e.g., flexibility, efficiency). This is the case for many companies that are experiencing productivity losses due to the lack of real-time visibility into the location of their critical assets and logistics units.

While the challenge of selecting the most suitable technology design for a specific use case is common to many companies, the market offers a portfolio of indoor Real Time Location Systems (RTLS) for tracking and tracing people, products, and equipment. These solutions predominantly rely on competing geolocation technologies, with “active” transponders (tags that require batteries to power the asset tracking devices) being the dominant choice. However, for managing dormant equipment, such as items used sporadically and then stored for extended periods, solutions requiring tag battery maintenance or replacement pose drawbacks. Conversely, the emergence of new RTLS based on “passive” Radio Frequency Identification (RFID) technologies is disrupting the market, offering tags that do not rely on batteries for power [5].

As a result, several companies have taken advantage of technological innovations in the field of passive RFID and developed RTLS readers accordingly. Impinj (Seattle, WA, USA) led the way in 2014 with its passive RTLS reader, the xArray reader (2D x, y), followed by RF Controls in 2017 and Zebra in 2020 (3D x, y, z). Despite notable performance enhancements over the years, the adoption of these systems remains limited. Moreover, the academic literature on passive RTLS is sparse, often incomplete, or outdated. Recent literature reviews indicate a predominant focus on passive RFID technologies for real-time tracking and monitoring applications in logistics and supply chain management [6,7], leaving passive RTLS with minimal or no coverage. Other studies in the field of RTLS are primarily conducted by solution vendors, raising concerns about result neutrality.

This gap in the literature raises several questions about the most appropriate design for asset tracking (physical and software infrastructure) and the actual performance versus that advertised by vendors of alternative RTLS solutions, including those based on passive RFID technologies. For instance,

- Can passive RFID be considered a serious competitor to established RTLS technologies in the market?
- What key features should be used for comparing indoor passive RTLS technologies?
- What is the realistic performance of passive RFID RTLS technologies?

### Objective of the Research

This research project aims to (a) propose an up-to-date typology of RTLS and (b) clarify the realistic performance of passive RTLS solutions. To achieve the first objective, we review both the academic and industry literature. To achieve the second objective, following a design science approach, we develop and test an RTLS prototype based on passive RFID technologies in two different industrial environments.

In Section 2, we explore the central role of IoT technologies supporting innovative applications in Industry 5.0, particularly those related to RTLS solutions. We conduct a review of the academic and professional literature to identify the knowledge gap related to passive RTLS.

In Section 3, we present an updated, synthetic comparative analysis of RTLS technologies to reflect the latest industry trends.

In Section 4, we introduce a case study to anchor our research problem, aiming to validate the performance of emerging passive RTLS systems. We follow a design science research methodology to develop our artifact (the prototype solution).

Finally, in Section 5, we conclude and open the discussion on security issues related to emerging passive RTLS.

## 2. Literature Review

### 2.1. Industry 4.0 to 5.0

As Garms [8] points out in a McKinsey report that draws insights from companies adopting Industry 4.0, “Succeeding in Industry 4.0 requires more than just using new IT tools; it demands a comprehensive tech-driven overhaul of operations”. This approach to digital transformation involves automating processes and integrating new technologies throughout the company’s value chain, linking different systems within the plant, and synchronizing them. Industry 4.0 aims to use digital technologies to enhance the efficiency of value chain activities [9], respond to the increasingly personalized needs of customers, and adapt more quickly to market changes. Building on this concept, Industry 5.0, as proposed by the European Commission [10] retains the technological aspect of 4.0 but emphasizes three pillars: (a) business agility and resilience supported by advanced technologies, (b) prioritizing humans in innovation and competitiveness by focusing on skills development, and (c) embracing environmental sustainability and considering the product life cycle. A recent systematic literature review by Valette et al. [11] underscores the rising importance of Cyber-Physical Systems (CPS) and IoT concepts in industrial research, with a strong emphasis on human-centricity. They advocate for viewing future industrial systems as complex socio-technical entities, where human and industrial assets are seen as interconnected. Galdysz et al. [12] conduct a systematic literature review on the transition to Industry 5.0 and find that the integration of the human factor is often overlooked. They suggest exploring the “grey” literature to enhance our understanding and recommend practical studies to implement technical concepts. To address these insights, our paper will (a) incorporate real-world deployments of RTLS in manufacturing contexts and (b) present a case study in the paper’s final section to provide a practical grounding. This approach aligns with Leng et al.’s [1] observation that few papers discuss the implementation journey of Industry 5.0.

### 2.2. IoT in the Industry X.0 Technology Portfolio

The Internet of Things (IoT) plays a vital role in enhancing business flexibility and resilience, serving as a key component of Industry 5.0 [1]. It creates a technological environment where every physical object, whether living or not, can communicate automatically and in real-time with its surroundings, managing transactions efficiently [13]. Each connected object becomes a cyber-physical device, contributing to a smarter ecosystem. De Paula Ferreira [14] identifies “smart products”, which are uniquely identifiable and constantly tracked items, as crucial for implementing digital transformations in Industry X.0.

Research on this topic has surged over the past two decades across various industries, with a particular focus on Industrial IoT (IIoT) systems [15], which support operational processes [8,16]. The interaction facilitated by IoT technologies between physical and virtual realms has led to the emergence of digital twins, where physical entities have real-time updated digital replicas [17,18] throughout their lifecycle.

**Figure 1:**
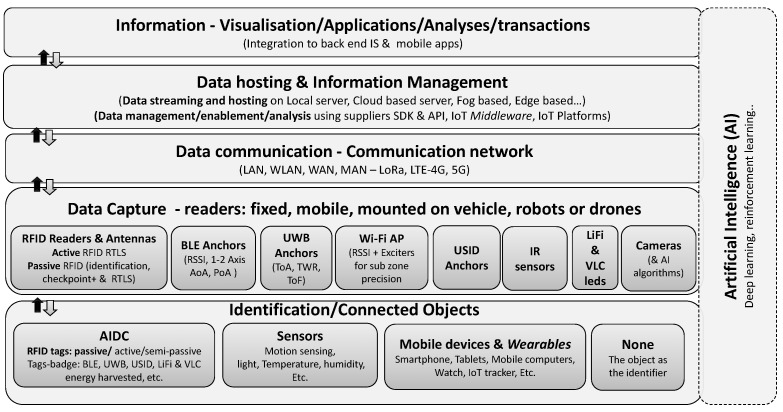
IoT multi-layer infrastructure: emphasis on RTLS.

Figure 1 shows the infrastructure of an IoT solution derived from various models proposed in the literature (adapted from [13]).

This model helps us to grasp that implementing an IoT solution involves the integration of multiple evolving technology layers based on various protocols and standards for (a) object identification and connectivity, (b) real-time data capture by readers, (c) wireless communication networks, (d) local or cloud-based data storage platforms, and (e) software platforms for visualization, analysis, and integration with back-end systems. AI (Artificial Intelligence) advancements over the past decade have integrated vision recognition solutions into this portfolio, where objects serve as identifiers recognized by algorithms. AI also enhances communication network performance and database management and primarily facilitates extensive data analysis and decision-making in various management systems.

Our research focuses on RTLS solutions within the (i) identification and (ii) data capture layers of an IoT infrastructure. These location-based functions are vital in the context of “smart factories”, [19] and IoT technology innovations have made implementing such systems more feasible, as seen in various offerings by technology providers. In a systematic mapping study, Haibi et al. [20] highlight a significant and ongoing increase in RFID research activities worldwide, particularly in supply chain and logistics management. However, only a small percentage of these studies address localization techniques. Despite their significance in the IoT realm, the academic literature shows limited interest in the design and development of localization solutions based on passive technologies [19]. Some studies [21] propose such solutions but tend to rely on multiple fixed readers rather than passive RTLS readers. Specifically, while RFID technology garners significant interest [6], deployment in logistics initiatives primarily focuses on tracking (41.67 % of the results) and monitoring (31.25%) applications [7].

### 2.3. Summary Analysis of RTLS in the Academic Literature

It is important to note that in the literature, Indoor Positioning Systems (IPS) typically refer to location-based services (LBS) on mobile devices, while RTLS generally focuses on locating objects using tags. Both terms are linked with indoor location-based services (ILBS), which emphasize both location and using processed data for decision making.

From academic and professional perspectives, various comparative analyses aim to assess the performance of RTLS/IPS, but very few consider passive RTLS as an option.

For example, in a recent survey of Indoor Location Technologies, Techniques, and Applications in Industry, Hayward et al. [22] summarize commercially available technologies. They use various criteria like cost, privacy/security, power consumption, coverage, scalability, accuracy, interference, and signal requirements. However, their analysis of BLE or RFID technologies is quite simplistic. They fail to break down the technologies and techniques to accurately represent recent market developments. Specifically, for passive UHF RFID technologies, they suggest coverage ranging from 10 m^2^ to over 1000 m^2^ with an accuracy of 1 m to 5 m.

Farsani et al. [23] propose an interesting survey on Indoor Positioning Systems for IoT-Based Applications. The authors discuss IPS classification, IPS algorithm classifications, and enabling communication technologies and discuss common metrics in IPS comparisons: accuracy (closeness of the measured or estimated position to the real one) and precision (closeness of a quantity in repeated measurements over time). Unfortunately, they do not include availability–accessibility of a service, cost, coverage, energy efficiency, latency, scalability, and robustness. Unfortunately, in their analysis, they completely omit the latest developments in passive RFID technologies.

Zafari et al. [24] addressed the need for an updated survey paper incorporating recent accurate and reliable localization systems. In their work “Emerging IoT Technologies-Based Localization”, they discuss Low Power Wide Area Networks (LPWANs) like LoRA, which require integration with other technologies for localization. However, their analysis of Passive RFIDs is oversimplified, indicating a “limited communication range (1–2 m)” and relying on prior research with fixed RFID readers.

Halawa et al. [25] narrow down RTLS technology choices to UWB, RFID, WiFi, and Vision. They specifically integrate vision-based object recognition systems; while they consider the “3D location” criterion achievable with various solutions, their assessment of RFID technologies is broad, grouping all types into one category.

The authors of [26] propose a comparative study on “Seamless Asset Location and Tracking Technologies”. They present an evaluation matrix comparing tracking approaches for both indoor and outdoor technologies. Although they differentiate between active and passive RFID technologies, their analysis overlooks the potential of the latest passive RFID technologies for localization.

Asaad and Maghdid [27] explored various metrics to compare localization solutions within the IoT landscape, including accuracy, precision, cost, scalability, latency, security, reliability, and system complexity. They rightly highlighted the complexity of indoor RTLS deployment compared to open-air settings. The authors mention that passive RFID technologies “are gaining popularity for localization due to their low cost, ease of installation, and maintenance”, but they do not elaborate on this discussion.

The authors of [28] examined RTLS applications in manufacturing to offer a comprehensive framework for positioning available RTLS solutions. Unfortunately, their discussion excluded passive RTLS from consideration.

Comparative analyses of RTLS technologies often fail to distinguish between evolving technologies. For instance, Bluetooth technologies now include BLE AoA (angle of arrival), BLE TDOA (time difference of arrival), and BLE RSSI (received signal strength indicator). Similarly, studies often lump together checkpoint RFID solutions and RTLS solutions equipped with multi-beam antennas.

The academic literature on passive RTLS is scarce, incomplete, or outdated, with most studies provided by solution vendors, raising concerns about the neutrality of the findings. The comparison presented in Table 1 outlines the characteristics of the referenced papers. Within the contribution column, each paper’s unique advantage is highlighted. The active RFID column denotes whether each paper addresses this technology. In the passive RFID column, the discussion of RTLS Passive UHF RFID specifics by each paper is indicated. Additional information is provided in the final column.

### 2.4. Summary Analysis of RTLS in the Professional Literature

#### The Established Active RTLS Technologies

In both the academic and professional literature, there is a wealth of information about active-based RTLS technologies, covering both current and emerging options. For instance, [29] from the *Sensors* journal discusses Wi-Fi as a promising option for indoor positioning, highlighting important positioning schemes and addressing challenges like the multi-path effect and data privacy. Another study by [30] explores the potential and limitations of BLE technologies, focusing on using RSSI measurement for indoor positioning and suggesting BLE AoA for future research. Additionally, [31] compares different wireless communication-based technologies for IPSs, particularly focusing on UWB-IPS for IIoT and emphasizing the characteristics of UWB technologies, such as large bandwidth and high data rate, crucial for precise indoor localization accuracy. However, they overlook passive RTLS in their analysis. On a different note, [32] delves into the advancements in IPS via visible lights, presenting it as a forthcoming low-cost solution for indoor localization.

In the professional literature, numerous documented cases showcase the deployment of these technologies across various business fields, demonstrating their effectiveness and versatility. There are also several IPS/RTLS comparative analyses proposed in the form of white papers, where each vendor promotes its technology as the best. For example, vendors like BlueIoT and Quuppa advocate for BLE AoA technology, emphasizing its high location accuracy, low power consumption, and compatibility with numerous BLE devices. On the other hand, UWB vendors like Ubisense and Sewio highlight the high accuracy and longer range of UWB technology. However, these reports often oversimplify the comparative analysis and neglect passive UHF RFID technologies.

**Figure 2:**
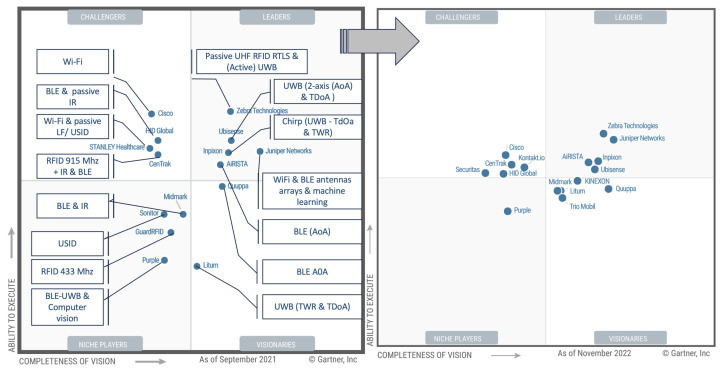
Adapted version of Gartner Magic Quadrant for indoor location services—2022 and 2023.

For a deeper market analysis, research and consulting service providers like Gartner offer comparative analyses of IPS, although they mainly focus on active technologies. Figure 2 presents an adapted version of Gartner’s “2022 and 2023 Magic Quadrant for Indoor Location Services” (adapted from [33,34]). The leading vendors are positioned in a two-axis map of (a) completeness of vision and (b) ability to execute. We modified this matrix to highlight the variety of competing technologies and positioning techniques in the market.

### 2.5. Actual and Emerging Indoor RTLS Options

One common approach is WiFi-based RTLS, utilizing antennae, proximity detection devices, and efficient algorithms for accurate location. Well-known vendors in this domain include Cisco, Aruba, and Securitas (formerly Stanley Healthcare).

Bluetooth Low Energy (BLE) has also made strides in RTLS. Many existing BLE-based solutions rely on received signal strength (RSSI), limiting accuracy to 4–5 m. Notably, vendors like HID Global and Minew operate in this space. Some, like Arista or Quuppa, employ BLE/AoA for more precise localization accuracy, reaching a few centimeters.

Ultra-wideband (UWB) is widely used in RTLS and is less affected by signal interference and capable of penetrating various materials. With localization accuracy ranging from 10 to 25 cm, UWB utilizes triangulation algorithms based on Time of Arrival (TOA), Time Difference of Arrival (TDOA), and more recently, Time of Flight (TOF). Zebra, Sewio, Juniper, and Ubisense are established vendors in this sector.

Ultrasound identification technologies (USID) offer reliable indoor tracking with centimeter-level accuracy, boasting energy efficiency and easy deployment. However, the speed of sound variation due to environmental factors affects signal performance. Sonitor is a prominent market leader, now enabling the geolocation of smartphones.

Visible Light Communication VLC and Light Fidelity (LiFi) using light sensors to measure the position and direction of LED emitters, while providing high accuracy, line-of-sight between the LED and sensor is necessary. Companies like Lumentrace combine VLC-based localization with Bluetooth communication.

Active RFID technologies, operating in various frequency bands (6–8 GHz, 2.4 GHz, 433 Mhz, 915 Mhz), face limitations due to standardization issues and the need for additional infrastructure for localization. Hybrid active RFID tags, operating on different frequency bands, employ sensors like infrared, low-frequency, or ultrasonic for precise identification in specific areas. Centrack for instance uses active 915 Mhz tags also equipped with infrared (IR) sensors for precise location.

The portrait of the industry therefore presents multiple technological options; nonetheless, it is incomplete. The inclusion of passive RTLS in this portfolio of solutions is now a must. There is therefore a need to update and validate RTLS technology categories in the academic and professional literature reviews.

While there is also a wealth of the academic and professional literature regarding passive RFID technologies (battery-less tags activated by electromagnetic emitted by the antennae), most documented cases of these technologies in business fields, like those found in *RFID Journal*, involve fixed or portable readers. In the case of RTLS, which relies on passive RFID technologies, tag localization is now made possible by the use of omnidirectional (multi-beam) phased-array antennas. This technology was first introduced in 2008 by Mojix and developed over the following years, still struggling to establish itself in the market. The first passive RTLS cases emerged in the retail sector after Impinj introduced the Power-over-Ethernet (PoE) phased-array antenna xArray ceiling reader in late 2014 [35]. This reader is equipped with 52 beams with both horizontal and vertical polarizations covering up to 1500 ft² (140 m²) within 1.5 m (5 feet) of an item’s actual location.

In 2017, RF Controls introduced the CS-445B passive RTLS reader antenna, featuring an overhead bidirectional, steerable array. Revealed at the 13th Annual RFID Journal Live, it won the Best New Product award. The first generation of the CS-445B model was designed for ceiling heights of 10 to 30 feet, with a 45-foot read range and the ability to locate tagged items with an accuracy of about 18 inches [36]. In 2020, RF Controls launched the CS-490 antenna, reading tags up to 50 feet high and reaching a maximum distance of 90 feet, suitable for warehouses and manufacturing plants. This version offers 3D positioning (x, y, z) and higher accuracy when multiple antennas are used. In open spaces, mounted at 30 feet, it can cover a 3600 ft2 scan area. RTV Engineering and RF Controls deployed the technology in a manufacturing company to identify, locate, and track materials stacked seven bins high on metal racks facing each other across an aisle. The deployment of five RF Control antenna units, at a height of 32 feet, covered a 100-foot aisle with 10-inch accuracy [37]. Recently, Tego, an Industrial IoT (IIoT) solution provider, partnered with RF Controls to deploy a passive RFID-based RTLS for aerospace and defense companies. The technology, installed 50 feet above the floor, accurately locates, tracks, and encodes passive UHF RFID tags attached to forklifts, returnable transport items, and assets, with an accuracy of 1 to 3 feet [38].

In addition to enhancing reading distance and location accuracy, it is worth mentioning the distance-encoding feature, which allows pinpointing a specific tag’s location for encoding, even from a distance of 500 feet [39]. Continuing in the same line of product development, Zebra Technologies, a leading RFID-IoT vendor, launched its ATR 7000 high-ceiling overhead reader in late 2019, enabling RTLS solutions with passive tags [40]. This reader works alongside their license-based RTLS software, CLAS (Configuration, Location Analytics Software), to visualize tag locations on a map. Zebra also introduced the ZBR4000 passive RFID tags, designed to work seamlessly with the ATR. One of the earliest deployments occurred at a US transportation company, enhancing real-time inventory tracking and facilitating warehouse cross-dock activities [41]. Zebra utilized this pilot project to enhance its “Motion Works” platform, providing visibility into operations and streamlining integration with existing warehouse management systems (WMS).

## 3. Redefining the Portfolio of RTLS Options

### 3.1. The Emergence of RTLS Based on Passive RFID

**Figure 3:**
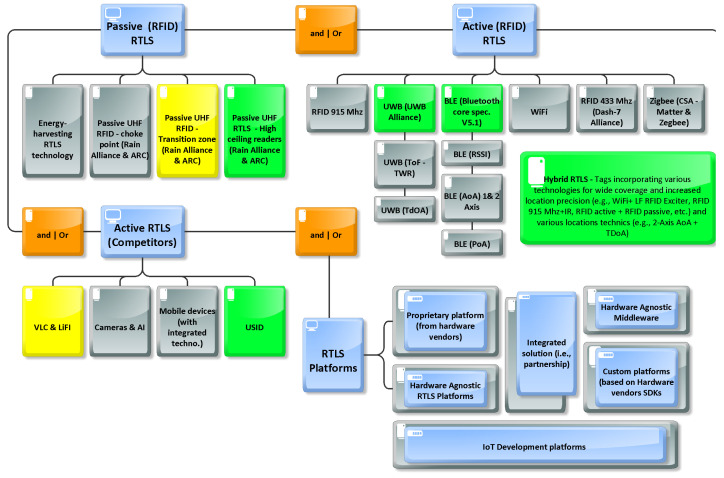
Updated indoor RTLS portfolio.

Based on previous analyses, we propose an updated portfolio of RTLS summarizing the different technologies used in RTLS solutions (see Figure 3).

This figure presents a current overview of the various technologies utilized for item identification and real-time tracking. The figure is segmented into four technological blocks. The first block represents the software infrastructure used to manage RFID-IoT data which is required whatever the choice of the hardware used to identify and locate items. Then, the next three blocs represent different types of hardware infrastructure; while these technologies often compete, they can also complement each other when integrated into a single solution, leveraging the strengths of each system. For instance, one technology might allow full zone coverage with low accuracy for detecting tagged items, while another technology would precisely identify the transit of tagged items between zones. These blocs are discussed in more detail below.

The first block “RTLS Platforms” concerns the software platforms used to manage RFID-IoT data gathered from the hardware infrastructure. In the case of active RFID solutions, there are several business models: (i) proprietary platforms, in which case the tag supplier also offers its own platform (e.g., Mobileview from Securitas, Quuppa Software suite or Simatic RTLS from Siemens), (ii) agnostic software platform providers whose solution enables the management of the data obtained, including businesses processes and workflow content, as well as dashboards and integration with management systems (e.g., Tego’s Asset Intelligence Platform-AIP; Catamaran NextGen from Shipcom), and (iii) more simplistic middleware enabling basic but crucial functions such as the acquisition, the processing, and the transfer of the data obtained to back end systems (e.g., Pareto Anywhere from reelyActive or Clearstream from PTS Mobile). IoT platforms from well-known vendors can also be used to develop robust RTLS solutions (e.g., Microsoft Azure IoT, Amazon AWS).

The second block “Active (RFID) RTLS” represents established active RFID technologies (such as BLE, WIFI, and active RFID), each employing various techniques. For instance, BLE technologies utilize methods like the received signal strength indicator (RSSI) for localization accuracy within a few meters, BLE Angle of Arrival (AoA) for sub-meter accuracy, and BLE AoA on two axes (y, z) for even greater precision. Additionally, BLE phase-ranging technologies extend battery life. It is worth noting that the trend towards hybrid tags, which combine features like wide coverage detection with precise localization, often incorporating ultrasonic sensors or low-frequency receivers. In another scenario, a tag could be equipped with a BLE tag to calculate location and employ low-power wide-area (LoRa) gateways to forward data to a server.

The third block “Active RTLS (competitors)” consists of other battery-equipped technologies competing with active RFIDs, including VLC (e.g., Lumentrace), LiFi, and USID (e.g., Sonitor), as well as cameras with AI algorithms and increasingly advanced detection capabilities in mobile devices.

The fourth block “Passive (RFID) RTLS”, of particular interest, encompasses the portfolio of passive RFID readers, which fall into four main families presented in the next section.

### 3.2. Key Players and Technologies in the Field of Passive RTLS

First, we find fixed readers used as checkpoint types (e.g., gates) that detect the presence of a tagged object when it passes within the radiation field of the reader’s antennas. In order to determine the direction of the tag, antennas must be installed or external devices (e.g., motion detector, laser) are required.

Second, there are movement detection readers (e.g., directional gates). These readers are equipped with at least two antennas that detect (a) the presence of a tagged object and (b) the direction of passage of the tagged object (e.g., Impinj xSpan gateway, Kathrein ARU8500 reader, and Zebra ST5500 Transition RFID Reader)

Third, “real” passive RTLS readers include Impinj’s xArray which offers the possibility of geolocating a tagged object with precise coordinates (x, y) in a ceiling of fewer than 15 feet. As discussed, more recently, other passive high ceiling RTLS readers equipped with Steerable Array Antenna from RF Controls (Smart Antennas) and Zebra (ATR 7000) which provide coordinates in 3D (x, y, z) are now redefining the landscape. A comparative matrix of such readers is shown in Table 2. In this table, various features are presented for comparing indoor passive RTLS technologies. For example, coverage indicates the area that antennas cover. Location accuracy indicates the level of precision and error in detecting the location of each object. The reading distance is the maximum readable distance from a tag to a reader. The location dimension shows the number of dimensions in each technology, which can be two-dimensional or three-dimensional.

Fourth, in parallel to the development of readers, one should note the development of energy harvesting tags. Indeed, while most passive RFID tags reflect back radio waves from the reader (responding), energy harvesting is a technique in which energy (from the reader) is gathered by the tag, stored briefly, and transmitted back to the reader–receiver (e.g., [42]). Uwinloc is an established provider of such solutions in the Industry 5.0 Context. Their solution combines (a) RFID UHF Exciters located near storage-detection areas (b), powering passive harvesting tags (c) which emit their unique ID back to the UWB receiver (d), connected to Wi-Fi or ethernet network (e), to send the data to a location engine processing unit.

With continued innovation and performance improvements, passive RTLS are then competing in a market traditionally reserved for active RTLS. The next logical step for researchers is therefore to verify the performance of such systems.

## 4. A RTLS Case Study in Manufacturing

This case study takes place in the manufacturing industry where many companies have developed their core competencies around product development and manufacturing, paying less attention to the logistics aspect of their operations. Consequently, these companies often encounter logistical challenges because they lack insight into their ongoing operations and the whereabouts of their crucial mobile equipment. To address these issues, many companies turn to Real-Time Location Systems (RTLS). However, due to the rapid growth of various Internet of Things (IoT) technologies, architectures, and platforms in recent years, choosing, developing, and implementing an Industrial Internet of Things (IIoT) system has become exceedingly complex and time-consuming. It requires extensive technical expertise, which poses significant barriers to entry into the market, especially for small and medium-sized enterprises (SMEs) [43].

### 4.1. Defining the Motivation on the Case

Company A is a manufacturing company specializing in the production of food packaging and containers. The company’s lack of real-time visibility into the location of hundreds of critical pieces of equipment (specifically, the molds used to make plastic containers) has a direct impact on operations planning, forcing the company to reschedule production.

#### 4.1.1. Definition of the Business Problem

Because molds can be stored for months, even years, before being placed back on a press to produce the appropriate range, the problem is not immediately reported but arises during production planning. Typically, after each use, the mold is placed in a wooden crate and stored in the warehouse until the next use. Although the mold’s location is recorded in the system (by scanning its bar code and location), the reliability of the information is sometimes compromised. For example, the physical location of these molds is sometimes not updated in the ERP system (molds moved without notification, stored in inappropriate locations, borrowed without notification, etc.). However, given the large number of molds and the size of the multi-zone, multi-row, multi-shelf warehouse, the search for a specific mold can take several hours to several days—sometimes without being found. As a result, the search for a mold wastes a lot of time, mobilizes employees in a non-value-added activity, causes production delays, leaves machines idle, and significantly reduces productivity. If a mold is not found, it also means re-manufacturing costs for a new mold.

#### 4.1.2. Design Science Research Approach to Build the Case

**Figure 4:**
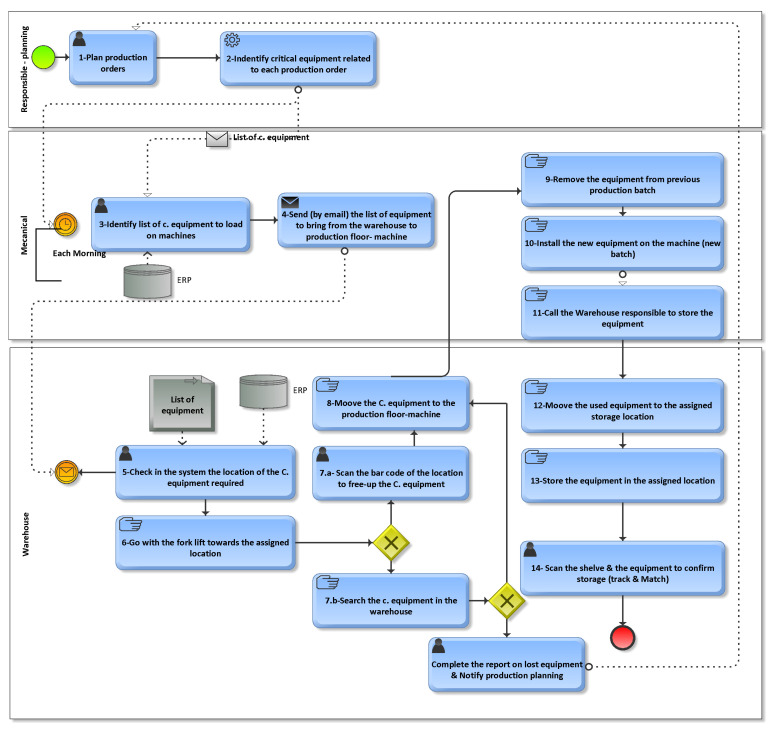
BPMN of the equipment management process.

**Figure 5:**
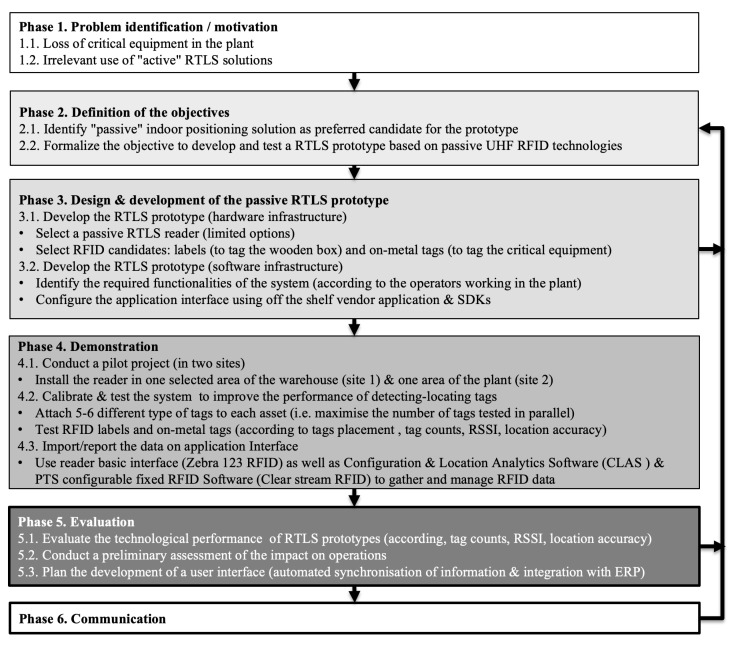
“Design Science” methodology used to build the case.

In this research, we adopted the “Design Science” methodology used in information systems projects, which focuses on solving practical problems through the design and development of an artifact, i.e., our passive RTLS prototype. This method proposes several stages, as shown in Figure 4 [44]. Given the scope of the pilot project, we have limited our approach to the first four steps of the methodology, see Figure 5.

### 4.2. Definition of the Pilot Project

To address this issue and ensure the visibility of this critical equipment, a “passive” indoor positioning solution is being considered.

#### 4.2.1. Objective of the Pilot Project

Given the defined context, the choice of such a solution must take into account several criteria: (i) require minimal maintenance of the tags since these molds can be stored for several months to several years before being reused; (ii) take into account the imposing structure of metal molds and their handling in the warehouse and the machines; (iii) ensure the detection of the molds everywhere in the areas of the warehouse equipped with 15 m high metal shelves; and (iv) be used in other possible use cases in the future, in particular for production monitoring.

Therefore, the objective of this pilot project was to develop and test a prototype solution for real-time location of critical equipment (e.g., molds) using the latest passive RFID RTLS available on the market that can (a) be deployed in large multi-shelf warehouses, (b) minimize maintenance management, (c) minimize implementation complexity, and (d) subsequently be used for production management monitoring.

#### 4.2.2. Key Criteria for Our Passive RTLS Prototype

Among the criteria selected for our RTLS application, which justify the relevance of a solution based on passive technologies, are (a) battery life, since molds can be stored for long periods of time before being reused, (b) localization accuracy of the order of one meter, which is sufficient to track a mold on a shelf, (c) the required reading range of 15–20 m from the ground, (d) 3D localization to identify molds on rows of multi-story shelves, (e) the complexity of installation, where we want to minimize cabling and the possibility of using RFID readers powered by POE (Power over Ethernet), with the possibility of relaying the captured information via WIFI, (f) the scalability of the system for use in other areas and/or items to be located, and (g) the total cost of the solution, including acquisition, installation and maintenance costs, installation, and maintenance costs—in this case, passive RFID RTLS solutions stand out for their low-cost tags and zero maintenance. Furthermore, the ability of the new RTLS readers to be installed in high ceilings makes it possible to cover large areas (30–40 m in diameter per reader), thus minimizing the number of readers required.

### 4.3. Design and Development of Passive RTLS Prototype

During the design and development of the solution (phases 2 and 3), it was first necessary to determine the desired functionalities of the RTLS system: (a) authenticate, (b) create/modify an item, (c) locate an item, (d) update the location of an item, and (e) display the location. This involves calculating the location of an item (x,y,z) and converting this spatial data into useful data for workers (row, shelf).

The IoT infrastructure used to build the RTLS prototype consists primarily of a physical data collection infrastructure based on Zebra’s ATR 7000 reader (Zebra Technologies: Lincolnshire, IL, USA) equipped with multibeam RF antennas that cover a 360-degree area. Because of the Zebra infrastructure already installed at the focal company and the familiarity of the employees with Zebra products, the ATR 7000 was chosen over the equally powerful RF Controls reader. Several tags were tested for identifying cases and molds. For cases, we chose Zebra’s ZBR4000 model for the crates (where the molds were stored) and Tag Factory’s M-Crown (on-metal tag) for the molds (The Tag Factory: Noida, India). Other tags from leading vendors such as Xerafy and Confidex were also tested and performed well. Once data are collected, they are transmitted over the local LAN communications network. The data are hosted in an SQL database to store the last location of each device and the various entities of our solution (SQLight DBMS included in the Django environment for the prototype versus the more robust MySQL or PostgreSQL for deployment). For the software infrastructure/application layer, the use of software development kits (SDKs) provided by the vendor enabled data acquisition and visualization. First, we used the CLAS (Configuration and Location Analytics Software V2.2.45.99) middleware provided by Zebra under license and hosted on a Linux virtual machine (running Ubuntu operating system 20.04) on a local server. This software is used to manage the readers, interpret the data they collect, and visualize the location of the tags on the floor.

### 4.4. Demonstration of the RTLS Prototype

#### 4.4.1. Testing the Passive RTLS Prototype—Site 1

**Figure 6:**
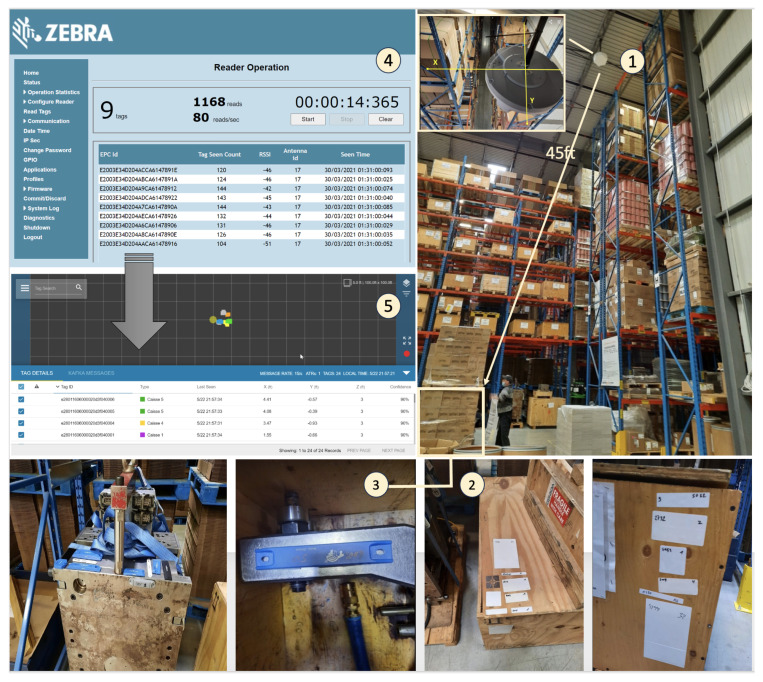
Deployment of ATR 7000 high ceiling passive UHF reader (from Zebra).

To test our prototype solution, we conducted a pilot project at the focal company’s warehouse. A specific area of the warehouse where the equipment is stored was chosen for the installation of the ATR7000 reader (Figure 6). The reader was mounted on the ceiling to cover the entire area and locate the tagged equipment (1). Selected RFID labels and on-metal tags were attached in various locations on the wooden boxes (2) and the metal molds (3). We were then able to test the (a) reading distance and (b) the reading reliability. For instance, regarding the reading distance, tag RSSI and tag count were used to ensure that the tags were still in the field of view of the reader (4). At this step, the tactic was to gradually reduce the power of the reader and observe which tags continued to respond. At lower power levels, only the “best-performing” tags, with the “best” tag placement were retained. For localization accuracy, the CLAS software was used to view (a) the tag movement on the 2D map and (b) the location accuracy (5).

The pilot project revealed that the range of this passive RTLS reader could reach an actual distance of up to 20 m radius (beyond the manufacturer’s specifications)—which means that the range of a reader could cover 40 m of a perimeter in an open space (with a reader 15 m above the ground). In terms of location accuracy, we found that the absolute coordinates returned by the CLAS middleware did not reflect reality. However, the relative position of a tag with respect to the reader and other tags was always correct. The value returned was stable and did not change with each reading, ensuring system reliability. This allowed us to obtain a fairly accurate location of the tools in our RTLS portal, clearly identifying a location column, but without being able to provide the floor (Figure 6).

**Figure 7:**
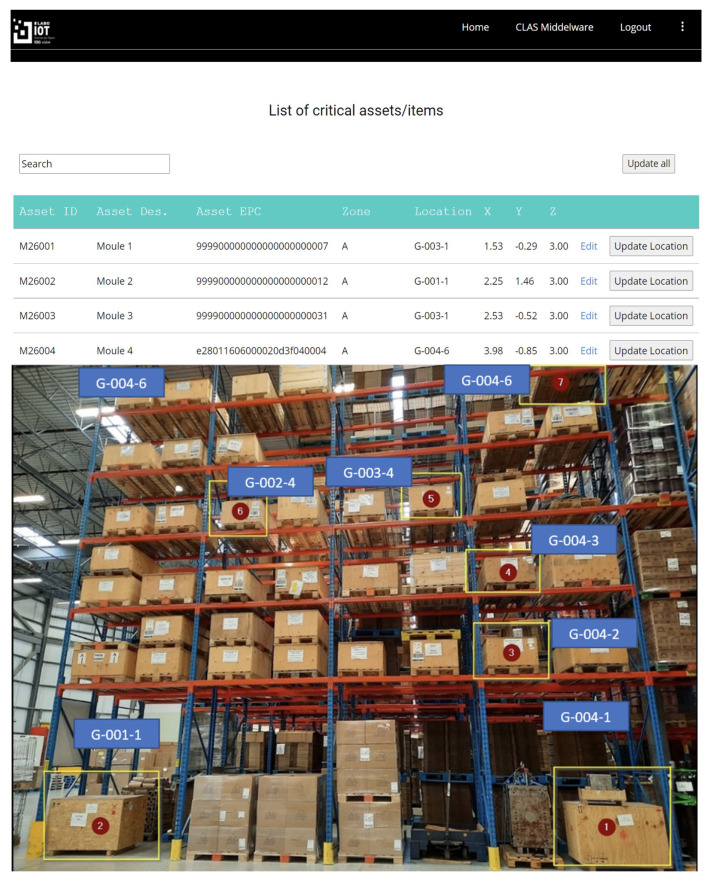
User interface reflecting the “real” location of the equipment.

From a user perspective, we then developed an RTLS application (back end) using the Python programming language to retrieve the raw data from CLAS (x, y, z coordinates) and convert it into more understandable and intuitive information for end-users (i.e., converted into location code). Finally, a web interface (front end) developed using web languages (HTML and CSS) was used to visualize the data provided by the application layer Figure 7.

#### 4.4.2. Testing a Passive RTLS Prototype—Site 2

**Figure 8:**
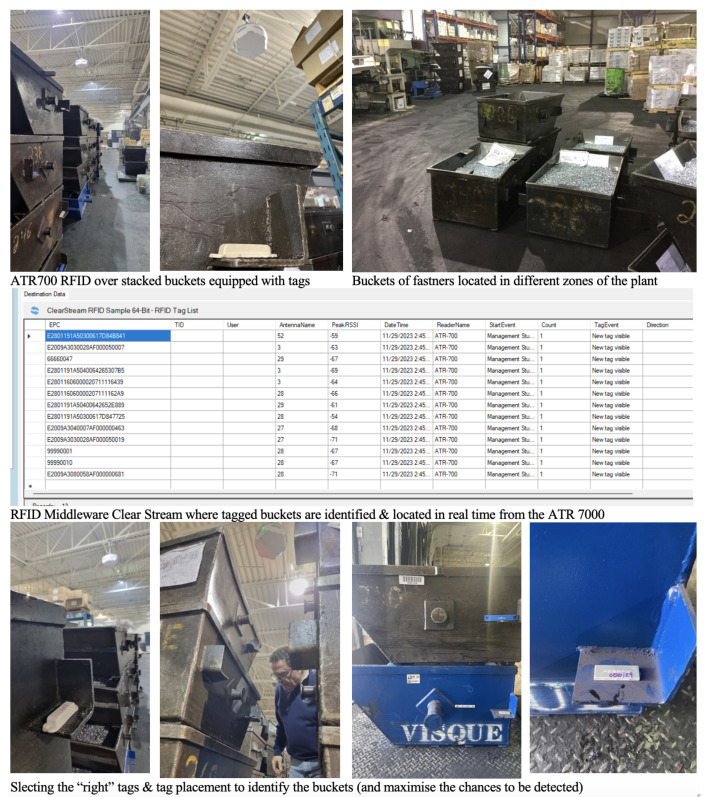
Deployment of ATR 7000 high ceiling passive UHF reader in another manufacturing facility.

From a technology performance perspective, although the deployment of our passive RTLS prototype was conclusive, we decided to continue the experimentation in another, more demanding environment. A pilot project was then conducted at the site of a North American manufacturer specializing in the production of fasteners. The manufacturer was struggling with a lack of visibility of work in processes tracking (WIP) of metal buckets used to transport production from zone to zone. Regarding the hardware infrastructure, we also installed an ATR 7000 from Zebra in the staging area where a great number of buckets are waiting to be processed (Figure 8). Regarding the software infrastructure, in this pilot project, all the data were managed using Clearstream RFID middleware v6.0 (Portable Technology Solutions, Calverton, NY, USA) a configurable fixed RFID and Bluetooth beacon software and that can be set up to track asset tags and integrate the data in the ERP of the company. Various information automatically gathered during the tests allows the researchers to assess the performance of each setting (i.e., Tag EPC, tag count, RSSI, date-time, tag-event, etc., Figure 8). To our surprise, in this pilot project, we realized that the tags tested in the previous case were not efficient at all. After various testing (tags, tags placement, reader configuration), we selected Xerafy (Versa Trak, Xerafy: Singapore) and HID (Exo InLine S, HID: Austin, TX, USA) to maximize the performance of the reads for locating and tracking the buckets.

The pilot project revealed that the range of this passive RTLS reader could reach an actual distance of up to 10 m radius (or 20 m of a perimeter) in an open space (with a reader 8 m above the ground). This was less than in the previous case, but this is mainly due to the installation height of the reader.

### 4.5. Evaluation of the RTLS Prototype

In this research project, we have demonstrated that our passive RTLS artifact provides a solution to our initial problem. The impacts are elaborated in the next sections.

#### 4.5.1. Passive RTLS: Evaluation of the Technological Performance

Although in both cases we were able to detect the tagged items, both pilot projects clearly revealed that passive RTLS deployments have to be customized based on each environment. Indeed, the choice of reader location and configuration, as well as the choice of tags, is clearly defined according to the products intended to be located and the operating environment. Both cases, and case 2 in particular, highlighted the interference caused by radio waves (reflection, diffraction) in metallic environments and the difficulty of identifying tags that are not in the reader’s line of sight. This effect can be attenuated by combining two readers, as recommended by passive RTLS vendors (vs one reader). This tactic would also have allowed for better coverage and detection of equipment in the blind spots observed during our pilot projects. However, due to the project’s budget limitations and the lack of availability of such readers in this market (still new at the time of the pilot), we were unable to obtain a second reader in time.

On the other hand, when it comes to tracking wooden boxes (case 1), the detection was much simpler, especially as special tags have now been developed for use in conjunction with high ceiling readers (i.e., Zebra ZBR4000 tag).

#### 4.5.2. Passive RTLS: Evaluation of the Impacts on the Operations

Getting back to case 1, from a process perspective, the solution we implemented automates the manual search for molds, significantly reducing the search time. In fact, an operation that used to take an average of eight hours (up to several days) is now completed in less than 15 min. Given that the cost of a production delay is estimated to be around USD 5000 per hour, this means that each mislocated part can cost up to USD 40,000. So, despite the relative precision of the proposed solution (in terms of shelf number), since the search process is limited to a very specific zone, the search time has been greatly improved. More interestingly, by tagging the storage boxes and the molds, the proposed solution solves put-away problems related to equipment assigned to a “wrong” box. This “untraceable” equipment, unless all the crates are opened, can force the company to re-manufacture a new mold, the machining cost of which is estimated to be around USD 85,000. With tags on both the crates and the molds, the location of the crates and molds (whose tags can be read through the wooden crates) is automatically known. However, to know if molds have been moved to another warehouse, the solution must be deployed in all warehouses, or at least at the warehouse entrances and exits.

From a user perspective, the next step is to have the users (warehouse workers) being able to take ownership of the technology and put it to practical use. This will allow them to evaluate the solution and identify possible gaps/improvements.

From a deployment perspective, the actual prototype has been tested in staging areas and shelving. It would certainly be useful to complement the real-time visibility of these critical assets by tracking them during the production life cycle (e.g., with a track-and-match application). Besides additional passive RTLS readers for wide coverage, fixed RFID readers for checkpoint detection could be used without extensive additional work since they use the same technology, protocols, standards, and platforms.

Finally, from an application point of view, the automated integration of location data with the ERP will make the process of finding equipment more efficient by eliminating the search task in our RTLS prototype. In addition, once the prototype has been validated—as is the case in our two projects—the diversity of IoT platforms compatible with passive RTLS systems greatly simplifies the deployment of such solutions. This should be a definite incentive for companies that decide to go ahead with such innovative projects.

Based on the discussed topics, it is observed that these systems have numerous advantages that could be further explored in future studies. For example, due to the lower cost of tags in comparison to other technologies, they are a suitable option for multi-ownership applications. This means that in a supply chain management system, where a product is transferred from one environment to another with different ownership and management, passive tags can still remain attached to each item. This feature provides current systems with greater flexibility. Therefore, studying how to manage passive systems and integrate them with existing systems is a suitable subject for future research.

## 5. Conclusions

This joint industry–university project is part of a very strong trend towards digital transformation, where RTLS deployments are still limited. It bridges a gap in the literature by providing a complementary and up-to-date perspective to the scientific literature on RTLS. In this work, we clarified the realistic performance of passive RTLS solutions and proposed a revised typology of RTLS. To achieve this goal, we developed and tested two passive RTLS prototypes using the latest technology available on the market. The pilot projects, conducted (i) in a multi-zone, multi-shelf warehouse location and (ii) in a harsh metal environment with stacked items, allowed us to test the potential and limitations of these latest passive UHF RFID technologies.

With continued innovation and performance improvements, there is no doubt that passive RTLS can be considered a competitor to active RTLS. This can be seen from several perspectives:

- Performance: coverage per antenna is increasing, and performance in terms of reading distance, reliability and location accuracy, low latency is constantly improving.
- Cost: the use of batteryless tags that allow for large-scale deployment with no battery replacement. From a total cost of ownership, (TCO), depending on the area to be covered, multiplying the number of readers can involve a significant upstream investment, but this initial cost can be absorbed by the very low cost of the tags and the maintenance.
- Deployment scalability: the ability of passive RTLS to be deployed in high-ceiling environments makes it suitable for warehouses and factories for both open areas and environments with shelves. In the latter case, however, it is important to ensure that the readers are positioned in the center of the aisles. The minimal configuration and calibration of the readers (vs active technologies), minimal cabling with POE power, and the use of WiFi to facilitate connectivity to servers simplifies the management of such projects.
- Integration: the availability of software SDKs, software applications provided by vendors facilitate the integration with existing systems, and partnerships with channel IoT platform partners are now possible to develop and deploy complete solutions.

Since passive RTLS is now a competitor to active RTLS, managers will have to build their business case accordingly and chose the most appropriate solution. On the other hand, managers can also consider passive and active RTLS as complementary solutions, leveraging the strengths of each system. Indeed, the wide coverage area and the scalability of active RTLS makes them a great option to locate mobile equipment (e.g., forklifts), while passive RTLS can be used to locate and track logistics units in specific zones. To identify the most appropriate RTLS, the decision should be based on the business case requirements including (a) the required functionalities of the RTLS solution, (b) the technical specifications derived from them, and (c) the project constraints.

That said, both pilot projects revealed that passive RTLS solutions are not as straightforward to implement, despite what vendors may suggest. Indeed, each new deployment requires full reevaluation of the RTLS design according to the physical environment.

In addition, to these previous considerations, we need to address another critical aspect of such IoT technology deployments in manufacturing environments and discuss passive RFID-based RTLS Security Concerns, while RFID-based RTLS has emerged as a powerful solution for real-time tracking and monitoring of various assets, objects and individuals, the widespread adoption of such systems also raises significant security concerns that must be carefully addressed to ensure the reliability and secure operation of these systems. The main security concerns associated with RFID-based RTLS are as follows:

- Unauthorized access and data interception: A major concern with RFID-based RTLS is the risk of unauthorized access to the system and interception of data in transit. Attackers may attempt to gain unauthorized access to the system, either physically or by exploiting vulnerabilities in the communication protocols, in order to collect sensitive location data or inject false information. Intercepting data in transit can compromise the confidentiality and integrity of the information.
- Tag cloning and spoofing: RFID tags used in RTLS systems often contain unique identifiers that enable accurate tracking and identification. However, malicious actors may attempt to clone legitimate tags or spoof their identities to gain unauthorized access or manipulate the operation of the system. This can lead to unauthorized tracking, manipulation of location data, or injection of false information, compromising the reliability and trustworthiness of the system.
- Denial of Service (DoS) attacks: The availability and reliability of RFID-based RTLS are highly dependent on the proper functioning of RFID readers, communication networks, and related infrastructure. Attackers can launch denial-of-service (DoS) attacks to disrupt the normal operation of these components, resulting in service interruptions, loss of tracking capabilities, and potential financial and operational consequences. The resilience of RTLS systems to such attacks is critical to maintaining uninterrupted and reliable tracking services.
- Data Integrity and Trustworthiness: Ensuring the integrity and trustworthiness of location data generated by RFID-based RTLS is paramount. Any unauthorized modification or tampering with location data can have serious consequences, such as misinformed decisions or compromised security. Therefore, data authentication, integrity verification, and tamper detection mechanisms are essential to ensure the reliability and trustworthiness of the information provided by the system

To address the above security concerns, several solutions have been proposed and are readily available. These solutions include a variety of approaches such as (i) disabling or “killing” tags to protect sensitive data, (ii) enabling “short-range mode” that allows a tag to respond only when the reader is in close proximity, and (iii) the use of various advanced security protocols (e.g., password) and techniques (e.g., Physically Unclonable Function (PUF)). However, these solutions are not yet readily available or suitable for passive UHF RTLS readers.

Potential technological advancements in Passive RTLS technology may include new tag designs for increased portability and application versatility. Integration of advanced sensors within tags could enable richer data collection, facilitating more precise tracking and environmental monitoring. Enhanced signal processing algorithms could further improve localization accuracy and reliability in complex environments. Additionally, advancements in communication protocols and network architectures could enhance scalability and interoperability, allowing for seamless integration with existing infrastructures. These advancements promise to revolutionize Passive RTLS technology.

Therefore, it is critical to carefully evaluate and weigh the pros and cons of each available solution to determine the most appropriate approach to address the specific security concerns in a given context. By weighing the pros and cons, organizations can make informed decisions to implement the most effective security measures while minimizing potential unintended consequences.

## Figures and Tables

**Table 1:** Comparison of academic related works.

Ref.	Contribution	Active RFID	Passive RFID	Additional Comments
[22]	Comparative analysis of indoor location-based services (ILBS): GNSS, Inertial System (MEMS), Wi-Fi, Bluetooth LE, WSN (Zigbee), UHF RFID (Passive), UHF RFID (Active), Ultra-Wideband, Acoustic (Ultrasound), Vision	YES	No	No consideration of BLE or passive RFID development
[23]	Comparative analysis of indoor positioning system (IPS): BLE5, WIFI, UWB, RFID Zigbee, VLC Acoustic, Ultrasonic, Vision, LoRa Sigfox and Cellular Comparative positioning techniques (RSSI, ToA, TDoA, RTT, etc.)	YES	No	No consideration of passive RFID technologies as an IPS candidate
[24]	Comparative analysis of Wireless technologies for Localization (IPS): IEEE 802.11 n/ac/ad/ah UWB, Acoustic, RFID’ Bluetooth, Ultrasound, Visible light, SigFox, LoRa, Weightless; Comparative localization techniques (RSSI, CSI, AoA, ToF, TDoA, RToF, PoA, Fingerprint)	YES	No	Active RFID “generic” No Consideration of passive RFID technologies as an IPS candidate
[25]	Comparative discussion on RTLS: UWB, RFID systems, vision systems, and Wi-Fi technology. RTLS technology evaluation	YES	No	Emphasis on UWB Technologies for RTLS. RFID is considered a tracking technology
[26]	Comparative Analysis among Tracking Approaches (RFID Active, RFID passive, BLE, UWB, Wi-Fi, LoRaWan, Cellular, GPS, Image)	YES	No	Segmentation of Active and passive RFID - where passive RFID is identified as short-range tracking technology vs RTLS
[27]	Comparative analysis of Localization Technologies and techniques GNSS (TOA, TDOA), Optical (TOA, AOA. TDOA, RSSI, Cell-Id), Wi-Fi (RSSI, RTT, TOA, TDOA, AOA, AP-Id), UWB(TOA, TDOA, RSSI, AOA), RFID (AP-ID, RSSI), Bluetooth (AP-ID, RSSI, TOA), FM, Cellular, Sound	YES	No	Point out passive RTLS developments but don’t discuss it
[28]	Qualitative comparison of RTLS-related communication protocols (UWB, Wi-Fi, BLEGPS, RFID, 5G)	YES	No	Consider passive RFID as a close-range technology

**Table 2:** Updated indoor passive RTLS.

	Impinj	Zebra	RF-Controls
Passive RTLS Reader	xArray	ATR7000	CS-445B Series and CS-490
Reader & steerable phased array antenna ETSI-Lower Band 865–868 MHz ETSI-Upper Band 915–921 MHz were allowed			
Coverage (sq. ft)	1500 sq. ft	N/A	10,000 sq. ft
Location accuracy (ft)	1–1.5	2	1.5–3
Reading distance (ft)	30 ft. radius	45 ft. radius	45–75 ft. radius
Distance encoding	No	No	Yes
Deployment - Mounting height (ft)	15	12–18	10–30/20–50
Power source	PoE	PoE+	PoE+
Location-Dimension	2D	3D (with 2 readers)	3D (with 2 readers)
Platform\OS	Linux	CLAS\Linux	RFC-OS
Directional readers (Zone monitoring and directional readers)	xSpan Gateway (1000 sq. ft)	ST5500 Transition RFID Reader	None
Other vendors: Kathrein Solutions ARU 8500 Antenna Reader Unit			

## Data Availability

Data are contained within the article.
